# Mitochondrial metabolic dysfunction and non-alcoholic fatty liver disease: new insights from pathogenic mechanisms to clinically targeted therapy

**DOI:** 10.1186/s12967-023-04367-1

**Published:** 2023-07-28

**Authors:** Youwei Zheng, Shiting Wang, Jialiang Wu, Yong Wang

**Affiliations:** 1 Department of General Surgery, The Fourth Affiliated Hospital of China Medical University, Shenyang, Liaoning Province China; 2 Department of Cardiovascular Medicine, The Fourth Affiliated Hospital of China Medical University, Shenyang, China

**Keywords:** NAFLD, Mitochondrial division, Mitophagy, Oxidative stress, Mitochondrial quality control, Lipid metabolism disorders, Targeted therapy

## Abstract

Metabolic dysfunction-associated fatty liver disease (MAFLD) is among the most widespread metabolic disease globally, and its associated complications including insulin resistance and diabetes have become threatening conditions for human health. Previous studies on non-alcoholic fatty liver disease (NAFLD) were focused on the liver’s lipid metabolism. However, growing evidence suggests that mitochondrial metabolism is involved in the pathogenesis of NAFLD to varying degrees in several ways, for instance in cellular division, oxidative stress, autophagy, and mitochondrial quality control. Ultimately, liver function gradually declines as a result of mitochondrial dysfunction. The liver is unable to transfer the excess lipid droplets outside the liver. Therefore, how to regulate hepatic mitochondrial function to treat NAFLD has become the focus of current research. This review provides details about the intrinsic link of NAFLD with mitochondrial metabolism and the mechanisms by which mitochondrial dysfunctions contribute to NAFLD progression. Given the crucial role of mitochondrial metabolism in NAFLD progression, the application potential of multiple mitochondrial function improvement modalities (including physical exercise, diabetic medications, small molecule agonists targeting Sirt3, and mitochondria-specific antioxidants) in the treatment of NAFLD was evaluated hoping to provide new insights into NAFLD treatment.

## Introduction

Over the past two decades, nonalcoholic fatty liver disease (NAFLD) has developed to rank among the most chronic and widespread hepatic diseases, and with the global obesity epidemic, its prevalence is increasing. It has become a serious threat to human health [1], because of the increased risk of comorbidities including cardiovascular disease, hepatocellular carcinoma (HCC), and type 2 diabetes (T2D) [2–4]. NAFLD is a metabolic stress liver injury closely related to insulin resistance (IR) and genetic susceptibility, with hepatic steatosis in the liver. However, other causes such as alcoholic liver disease, autoimmune hepatitis, drug damage and hypothyroidism are excluded. Genome-wide association studies (GWAS) have shown that changes in genes such as patatin-like phospholipase domain-containing 3 (PNPLA3) and transmembrane 6 superfamily member 2 (TM6SF2) have a differential impact on the development of NAFLD [5].The liver has an indispensable function in balancing lipid metabolism by modulating the production, oxidation, and transport of triglycerides (TGs), free fatty acids (FFA), cholesterol, and bile acids (BA) [6]. Excessive fat accumulation in the liver includes multiple conditions associated with the emergence and hepatic steatosis progression, these range from simple non-alcoholic fatty liver (NAFL) to complicated non-alcoholic steatohepatitis (NASH). NAFLD is not only closely related to liver cancer, but also involved in many extrahepatic diseases, such as bladder cancer and sarcopenia. Among them, the occurrence of bladder cancer is largely due to insulin resistance. A retrospective study shows that most patients with bladder cancer suffer from NAFLD, insulin resistance and T2D [7]. In addition, NAFLD is also closely related to sarcopenia, which we will focus on below. The excessive fat accumulation in Metabolic Dysfunction-Associated Fatty Liver Disease (MAFLD) patients, produces cytotoxic lipid oxidation byproducts that can cause NASH with a chronic necro-inflammatory state [8]. Notably, impaired mitochondrial metabolism is extensively associated with NAFLD by mediating the dysregulation of lipid metabolic homeostasis. With further research, the exclusive diagnosis of NAFLD can not meet the clinical needs, which is not conducive to early diagnosis and early intervention of diseases. Therefore, to reflect the hepatic manifestations of this metabolic disorder, the name NAFLD has been reconsidered and recently renamed MAFLD [9–11]. MAFLD is more likely to be obese and overweight.

The mechanism by which mitochondrial function regulates liver metabolism and therefore, interferes with the disease progression has been intensively investigated. Several studies have shown that factors such as mitophagy, oxidative stress, differentiation, and quality control, differentially influence mitochondrial function to promote liver fat accumulation and injury. These steps in part mediate impaired lipid metabolism to exacerbate the development of NAFLD. In patients with MAFLD, mitochondrial dysfunction-mediated disruption of lipid metabolism leads to excessive accumulation of TGs (> 5%) in hepatocytes and hepatic steatosis [12, 13]. MAFLD covers a range of diseases from NAFL with no obvious inflammatory manifestations to NASH, these hepatic steatoses are linked with lobular inflammation, pericyte fibrosis, and apoptosis, and cannot distinguish histologically from alcoholic steatohepatitis [14, 15]. However, the transition from NAFL to NASH is not only based on steatosis but is also driven by mitochondrial dysfunction characterized by dysregulated oxidative phosphorylation (OXPHOS) and reactive oxygen species (ROS) generation [16, 17]. Therefore, mitochondrial function regulation appears to be a potential strategy to stop the progression or even treat MAFLD.

Because of the underlying importance of mitochondrial metabolic disorders in NAFLD development, it is crucial to target its metabolic regulatory mechanisms for therapeutic strategies against NAFLD. The available literature suggests that physical exercise, antidiabetic drugs, and antioxidants may have the potential to reverse mitochondrial metabolic disorders and hold good promise for large-scale clinical application. Overall, this review focuses on the impact of mitochondrial metabolic dysregulation and lipid accumulation on the progression of NAFLD and discusses multiple potential therapeutic strategies to improve mitochondrial function, hoping to provide new insights for clinically targeted therapies based on NAFLD.

In reviewing the relevant literature, we drew on the approach of Baethge et al. to develop the following methodology for the literature search [18]. The first step is to retrieve the corresponding subject and free words in the corresponding database, using the logical operators “AND” and “OR”, the wildcard character “*”, etc. to connect the subject and free words. Also note the field identification of the corresponding phrase in the search box to limit the search and compose an advanced expression for advanced searching. The second step is to import the searched documents into the literature management software and subsequently read the title, abstract and, if necessary, the text in the literature management software to filter the required documents. If there is a large amount of literature, the relevant literature from the last 5 years was chosen.

## Lipid accumulation promotes MAFLD progression

Recently, multiple theories have been put forth to elaborate on the occurrence and development of MAFLD. The “multiple-hit” hypothesis has replaced the “double-hit " hypothesis of NASH, the former takes into account the promotion of MAFLD by multiple impairments in genetically susceptible individuals, such as hormones secreted by adipose tissue, insulin resistance, nutritional factors, and gut microbiota [19, 20]. Adipose tissue stores excess food calories in the form of TGs, and the accumulation of TGs and FFA in the liver is the result of dysregulated fat metabolism [21]. Excessive caloric intake may induce MAFLD/NASH by mediating gut microbiota imbalance and increasing portal circulation of bacterial products, causing activation of the natural immune system [22, 23]. Due to the enhanced circulating plasma FFA, IR occurs in the muscles of the body, which is a key step in MAFLD development [24, 25]. Furthermore, IR increases hepatic De novo lipogenesis (DNL) and stimulates adipose tissue to secrete adipokine and inflammatory cytokine, ultimately leading to the dysregulation of adipose tissue’s lipolytic processes [26]. Interestingly, fat accumulation in the liver increases lipotoxicity, causing an increase in oxidative stress which can severely impair normal mitochondrial metabolic programs and cellular functions in the liver and provide the potential for FA accumulation [27]. Therefore, this positive feedback loop (FA accumulation- IR-impaired mitochondrial metabolism -FA accumulation) may support the continued progression of MAFLD/NASH and establishes the possibility for the development of certain malignant diseases such as HCC.

**Fig. 1:**
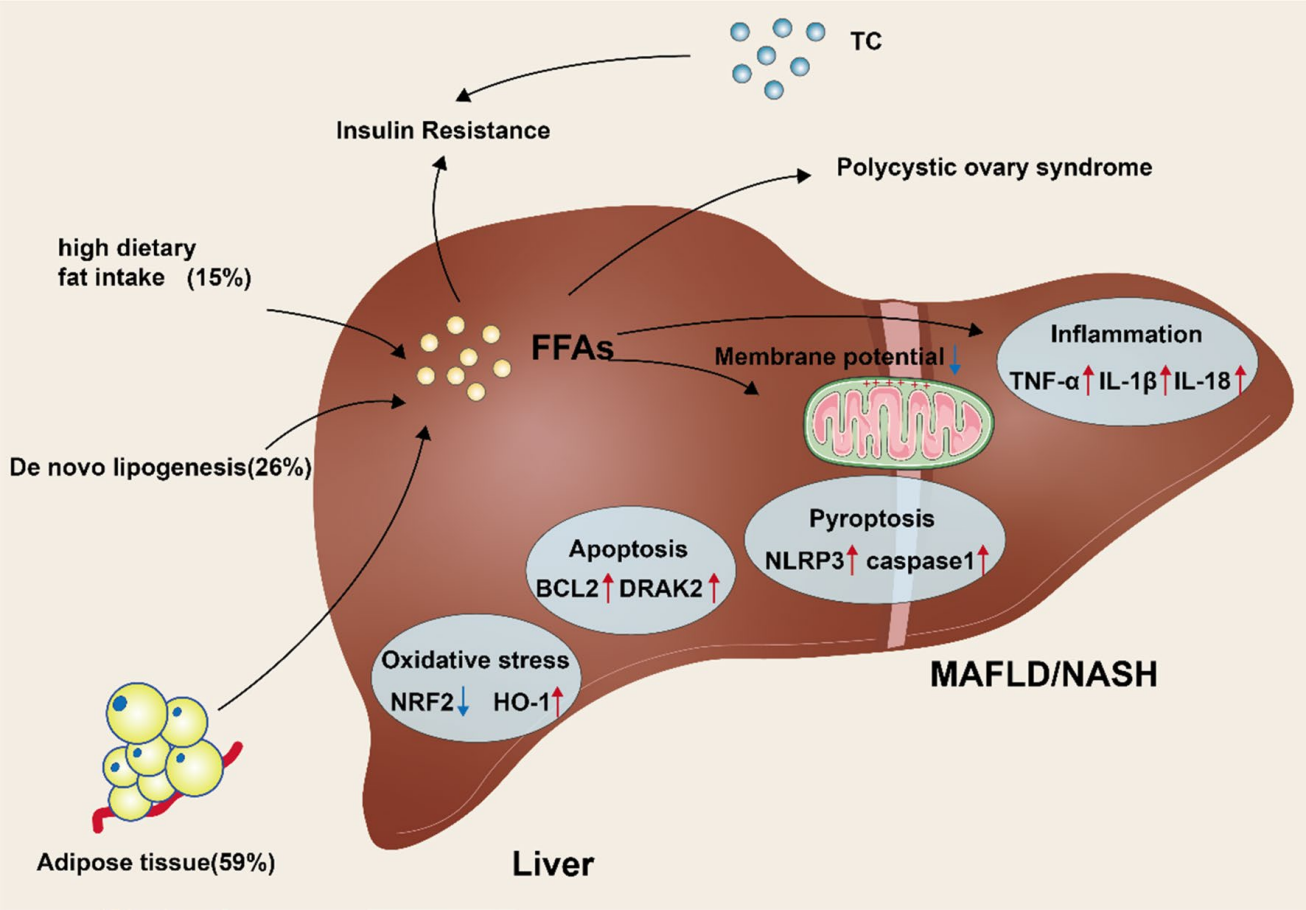
Diagram of the mechanism of MAFLD/NASH occurrence, and the various metabolic problems that occur along with it. High calorie foods, and the ab initio synthesis of fats lead to the production of large amounts of free fatty acids in the liver, which, together with excess total cholesterol, leads to insulin resistance in the body. High levels of free fatty acids can lead to a range of problems in the body such as polycystic ovary syndrome, oxidative stress, inflammation, apoptosis, pyroptosis and a decrease in mitochondrial membrane potential, ultimately leading to the development of NAFLD/MAFLD in the body

During MAFLD, FAs from different sources (such as dietary, DNL, and lipolysis) are utilized in hepatocytes to produce TGs, contributing to liver fat accumulation [28, 29]. Approximately 59% of the FAs that accumulate in the liver are sourced from adipose tissue lipolysis, 26% is from DNL and 15% is from the diet (Fig. 1) [28]. FFA can also be transformed into TGs and transported as very low-density lipoproteins (VLDL) [30]. This metabolic dysfunction mediates the accumulation of liver fat and causes the continued development of MAFLD. Interestingly, increased circulating levels of FFA and TGs and adipose tissue stored TGs also contribute to peripheral IR [31, 32], while persistent hyperglycemia and compensatory hyperinsulinemia contribute to the progression of T2D and fatty liver in obese patients. Indeed, FA accumulation mediates liver damage and fatty liver development in different dimensions. The ongoing fat metabolic breakdown related to IR causes elevated blood circulating FFA levels, which are readily transported to the liver and provide the conditions for fatty liver development [31]. Similarly, high carbohydrate consumption increases hepatic lipogenesis, thereby supporting fatty liver progression by increasing the synthesis and uptake of FFA [33, 34]. Notably, sustained accumulation of FFA may induce liver injury by activating immune-inflammatory pathways. FFA promotes nuclear factor-κB-dependent TNF-α expression by mediating lysosomal instability and stimulating increased hepatotoxicity to impair normal hepatic metabolic function [35].

It has been suggested that an abnormally high DNL could potentially contribute to the development of MAFLD [36]. A high carbohydrate diet supports NAFLD progression by inducing DNL to regulate the sterol regulatory element binding protein 1c (SREBP1c) expression [37, 38]. The development of NAFLD is accompanied by severe mitochondrial metabolic disruption and therefore, contributes to lipid accumulation to support NAFLD progression. For example, impaired mitochondrial metabolism in NAFLD impairs FA oxidation processes (e.g., mitochondrial beta-oxidation) and induces elevated DNL, which ultimately continues to drive NAFLD progression in the form of lipid accumulation [39, 40]. Moreover, when mitochondrial beta-oxidation is impaired, peroxisomal and cytochrome oxidation of FFA alternate, resulting in high ROS levels and dangerous oxidative by-products that support disease progression [41, 42]. Ma et al. [43] revealed that initially, the increased hepatic lipid availability in obese individuals with NAFLD enhances hepatic mitochondrial competence, however, it eventually stimulates enhanced hepatic oxidative stress and therefore, decreases mitochondrial activity, promoting the development of NAFLD into NASH [44]. Studies have also demonstrated that dysfunction in liver mitochondria precedes NAFLD and IR onset in obese rodent models [45]. Additionally, the endoplasmic reticulum (ER) is a crucial part of the stress response, it regulates a distinctive set of intracellular pathways, a phenomenon collectively referred to as the unfolded protein response [46, 47]. Interestingly, nuclearfactorkappa-B/calcium release-activated calcium modulator 1 (NFκB/Orai1) stimulates the regulation of the ER in intracellular pathways via oxidative stress to support the development of NAFLD [48]. Multiple NAFLD pathogenic mechanisms should not be viewed independently, as there may be ongoing crosstalk between different tissues or organs, where an intrinsic association of adipose tissue and the gut may contribute to NAFLD [49] because dysregulated intestinal metabolism contributes to fat accumulation and lipid deposition in the liver via the gut-adipose tissue-liver axis and mediates hepatic disease development [50]. Additionally, NAFLD pathogenesis is also influenced by genetic and epigenetic factors (including DNA methylation and histone modifications), with heritability estimates ranging from 20 to 70% [51, 52]. Specifically, activator protein-1 (AP-1) and early growth response (EGR) reprogram Kupffer cell properties and LXR functions required for survival to transform the associated macrophage phenotype and drive myeloid cell diversity in NASH [53]. As such, the known information suggests that epigenetic reprogramming may contribute to the phenotypic transformation of immune cells and favor the development of NAFLD by influencing their inflammatory environment [54, 55].

The elucidation of different mechanisms of disease progression facilitates the development of targeted therapies, while the intrinsic linkages in disease mechanisms are equally noteworthy, as their interactions pose serious challenges for clinical treatment. Therefore, understanding the interaction between different mechanisms is also beneficial for the clinical application of NAFLD-based targeted combination therapy.

## Mitochondrial metabolic dysfunction and MAFLD

### Mitochondrial division

The liver is one of the most mitochondria-rich organs, as the density of mitochondria depends mainly on the energy metabolic demand [56]. In hepatocytes, the mitochondrial activity includes metabolic pathways and signaling networks, which depend on mitochondrial DNA (mtDNA) integrity, affinity and antioxidant balance, membrane composition, transport of lipoprotein, and metabolic demand and supply [57]. For example, a case-control study assessed epigenetic changes in mtDNA, and mitochondria-encoded NADH dehydrogenase-6 (MT-ND6) hypermethylation was determined in liver biopsy tissue from MAFLD patients and correlated with the severity of histological disease [58]. These alterations are associated with ultrastructural changes in the mitochondria, manifested by inner mitochondrial membrane (IMM) loss, deep ridges folding, and mitochondrial granules loss, although these changes may be reversed by exercise [58]. The structural stability of mitochondria is essential for the maintenance of normal physiological activity. For example, exosomes released by Trem2-deficient macrophages can exacerbate NAFLD by damaging the mitochondrial structure of hepatocytes. The specific reason for this is because high levels of miR-106b-5p block Mitofusin 2 (MFN2) [59]. Similarly, the protein components of mitochondria-associated endoplasmic reticulum membranes (MAMs) are involved in the development of many diseases. Drp1 is a key factor in the control of mitochondrial division. In the striatum of Huntington’s chorea (HD) mice, overactive Drp1 activity is able to induce mitochondrial fragmentation, forcing mitochondria away from the endoplasmic reticulum and leading to the destruction of the MAM [60]. MAM proteins also include PTEN-induced putative kinase 1 (PINK), sigma-1 receptor (S1R), presenilin-1 (PS1) and so on. The above proteins are involved in Parkinson’s disease (PD) and Alzheimer’s disease (AD) respectively.In addition, genetic variants and metabolic stressors can stimulate mitochondrial dysfunction [61]. mtDNA mutations, particularly copy number variants and somatic point mutations, and mitochondrial metabolism abnormalities have been observed in HCC associated with NAFLD [62]. In this case, mitochondrial functions such as mitotic-nuclear communication and mitochondrial-ER contact (MERS) are impaired due to structural disruption, and this phenomenon may confer resistance to apoptosis and trigger the development of HCC [63, 64]. In addition, there is an association between impaired mitochondrial function and the development of sarcopenia. Mitochondrial dysfunction with impaired proteostatic mechanisms has been reported as an important factor in sarcopenia [65]. Interestingly, a reduction in muscle mass may contribute to the development of IR. Together with obesity, chronic inflammation and vitamin D deficiency, these factors are involved in the pathophysiological mechanisms of NAFLD [66]. Therefore, improving mitochondrial function can effectively alleviate sarcopenia and NAFLD. Maintaining the stability of mitochondrial structure and function and facilitating a complex balance between physical contacts between organelles and regulatory mechanisms at multiple levels, is also an essential basis for ensuring cellular homeostasis.

**Fig. 2:**
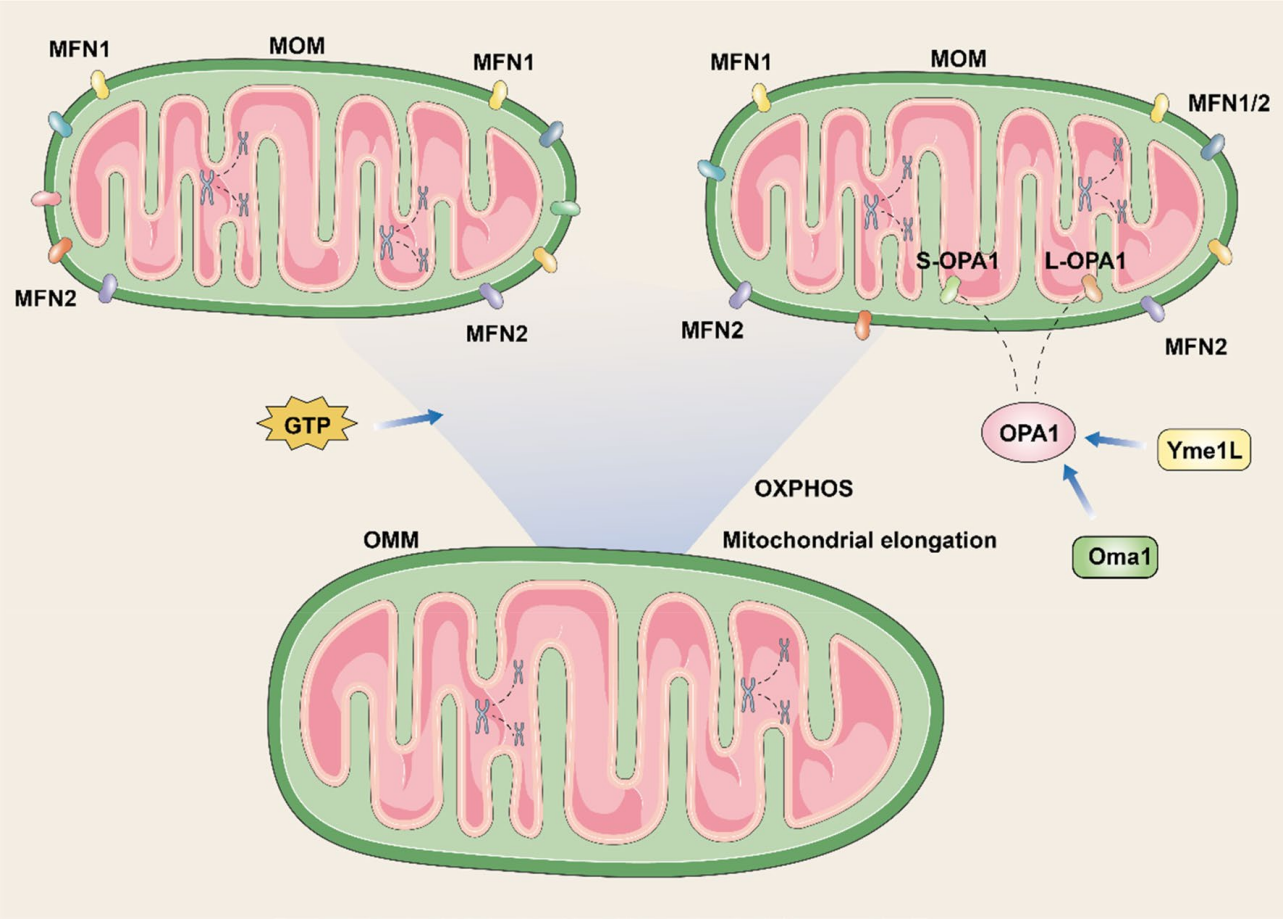
The process of mitochondrial fusion. Mitofusin 1 and Mitofusin 2 coordinate mitochondrial fusion, giving higher oxidative phosphorylation and mitochondrial elongation. Optic atrophy 1 is disassembled into S-optic atrophy 1 and L-optic atrophy 1 by the action of Oma1 and Yme1L, thus participating in mitochondrial connection and the formation of mitochondrial cristae

The length of the continuous mitochondrial renewal and degradation cycle determines the total number of mitochondria in the cell. During mtDNA replication, new mitochondria are produced from pre-existing ones when the transcription and translation processes of the nuclear and mitochondrial encoded genes are coordinated. Because mitochondria cannot be produced ab initio, cells use the alternating fusion-division steps in the mitochondrial pool to achieve its turnover [67, 68]. Mitochondrial fusion is usually associated with increased OXPHOS function and mitochondrial elongation, whereas, the main mitochondrial fusion proteins are Mitofusin 1 (MFN1)、MFN2 and optic atrophy 1 (OPA1), which are also required to maintain normal mitochondrial function. Specifically, MFN1 and MFN2 are widely distributed on the outer mitochondrial membrane (OMM), but they have different activities within the cell (Fig. 2). MFN1 is shown to have an 8-fold higher GTPase activity than MFN2 and mediates the fusion of the OMM in a guanosine triphosphate (GTP)-dependent reaction with a higher affinity for mitochondrial bridging [69]. OPA1 is crucial for the attachment of IMM with mitochondrial cristae formation. ATP-dependent Yme1L and Oma1 proteases regulate the cleavage of OPA1 into L-OPA1 or S-OPA1 [70, 71]. Depletion or abnormal activity of proteins mediating OMM fusion may lead to the emergence of defective mitochondria, which are then removed by autophagy. For example, uncontrolled Oma1-dependent cleavage prolongs the dissipation of mitochondrial potential, a phenomenon that leads to the complete inactivation of OPA1 and consequent disruption of mitochondrial structure [68].

**Fig. 3:**
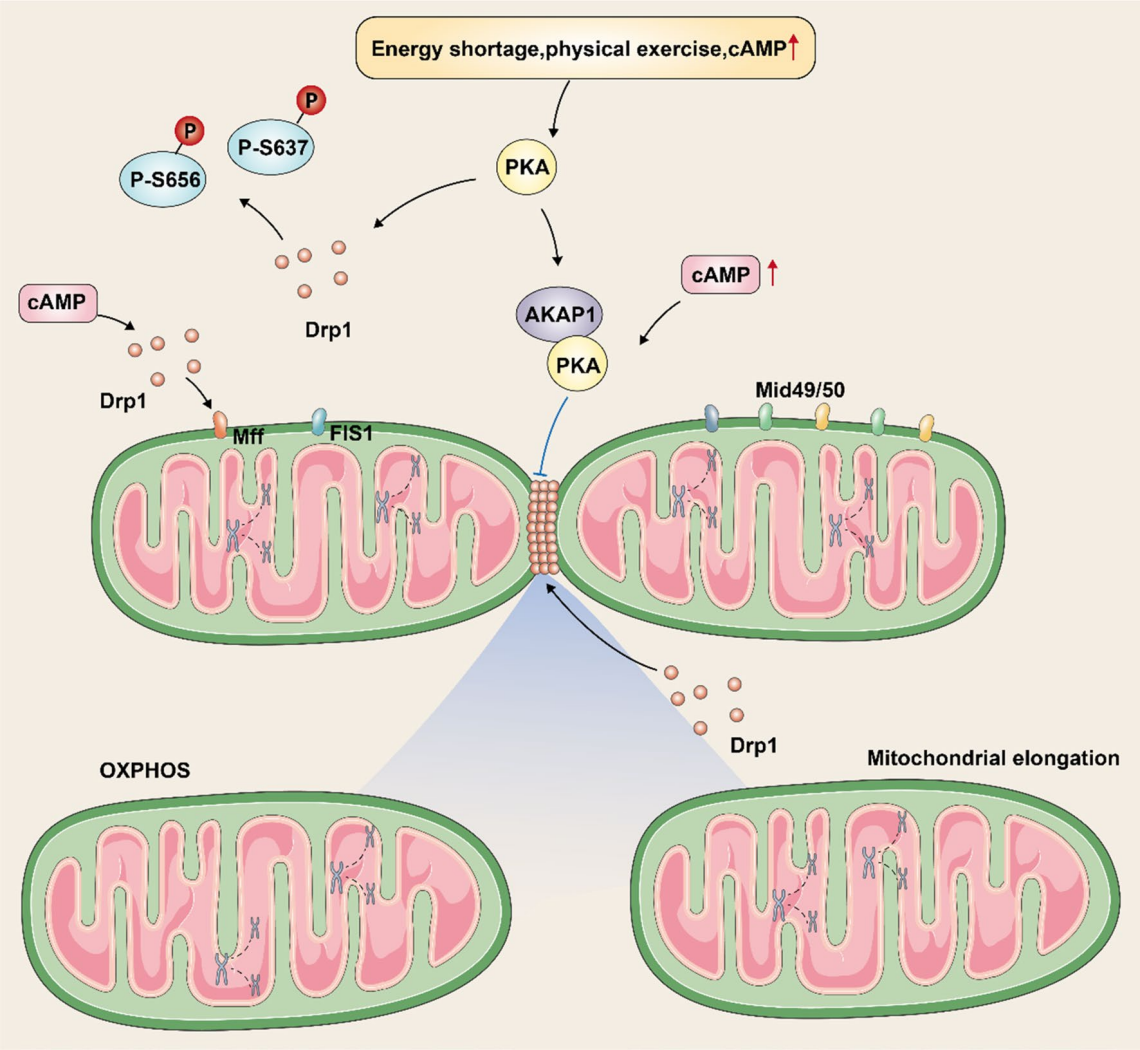
The process of mitochondrial division. mitochondrial fission factor and Mid49/50, as well as dynamin-related protein 1, are the main triggers of mitochondrial fission. Among them, dynamin-related protein 1 aggregates around the mitochondrial membrane to form a helical homopolymeric complex, followed by contraction of the complex causing mitochondrial fracture. Among them, energy shortage, physical exercise, and high levels of cyclic adenosine monophosphate lead to phosphorylation of dynamin-related protein 1 residues S656 and S637 by stimulating protein kinase A, which inhibits mitochondrial membrane breakage. Besides, high concentration of cyclic adenosine monophosphate can promote a kinase anchoring protein 1 binding to protein kinase A, and the formed complex inhibits dynamin-related protein 1 aggregation in the mitochondrial membrane and hinders mitochondrial membrane break

Dynamin-related protein-1 (Drp1), which is mediated by mitochondrial fission protein 1 (FIS1), mitochondrial fission factor (Mff), and the mitochondrial dynamin 49/50 receptor, and is recruited and activated around OMMs, is the main trigger for the cytokinesis process (Fig. 3) [72]. Activation of Drp1 may drive cell division in different dimensions and impair normal mitochondrial metabolic functions. Interestingly, post-translational modifications, energy insufficiency, physical exercise, and enhanced cyclic adenosine monophosphate (cAMP) stimulate protein kinase-A (PKA), which phosphorylates serine 656 (S656) and S637 residues of Drp1, thereby inhibiting the activity of Drp1 on mitochondrial damage in cytokinesis and offers the possibility of delaying the progression of MAFLD [73]. Research suggests that high cAMP concentrations favor co-localization and anchoring of PKA and Drp1 on OMMs via the protein scaffold A-kinase anchoring protein 1 (AKAP1) and Mff respectively, while the PKA-AKAP1 complex inhibits Drp1 and the fission process [74, 75]. In contrast, calcium-regulated neurophosphatase, a calcium ion/calmodulin-dependent phosphatase, promotes the formation of a helical homopolymer complex of Drp1 to wrap the mitochondrial membrane by reversing the phosphorylation of S637 and Drp1, which gradually contracts and induce mitochondrial rupture [76]. In liver cells, redox status, energy stress, reduced ATP, and NADH may stimulate the division of mitochondrial via the Sirtuin1 (SIRT1)/cAMP-dependent AMP-activated protein kinase (AMPK) signaling pathway [77], which regulates Mff phosphorylation resulting in Drp1 functioning on OMMs. However, in the muscle and liver tissues, during autophagy, a cascade reaction via cAMP/PKA/AMPK can phosphorylate Drp1 and prevent mitochondria from dividing [78, 79], which may be attributed to autophagy against cell division. In general, Drp1 activity is closely related to mitochondrial function, and specific means to modulate Drp1 expression or activity may be a viable strategy to rescue mitochondrial dysfunction and treat MAFLD.

### Mitophagy

The selective elimination of dysfunctional mitochondria by autophagic vesicles is called mitophagy [80]. Recognition of damaged mitochondria by autophagic vesicles is performed in a ubiquitin-independent and dependent pathway by LC3 adapters [81]. The PTEN-induced PINK1/Parkin pathway is the most researched mitochondrial autophagic pathway. In healthy mitochondria, progerin-associated rhodopsin-like (PARL) proteases and matrix processing peptidase (MPP) degrade PINK1 [82]. However, on the impaired mitochondria, PINK1 assembles and stimulates the elimination of these mitochondria in a docked manner [82]. The ubiquitin kinase PINK1 phosphorylates ubiquitin and activates the ubiquitin ligase Parkin [81]. GTPase mitofusin 2 on OMM is thought to regulate Parkin assembly in impaired mitochondria. PINK1 phosphorylates Mfn2 and promotes its parkin-mediated ubiquitination [83]. Parkin ubiquitinates proteins of OMM and facilitates their cross-talk with phagosomal adapters including NBR1, p62, and HDAC6 [81]. These junctions have LC3 interaction region (LIR) motifs, and LC3 can recognize and recruit labeled mitochondria into the autophagosome. In the mitophagy receptor pathway, receptors including Bcl2/Adenovirus E1B 19 kDa and Nip3-like protein X (Nix) and interacting Protein 3 (BNIP3) were found to bind directly to LC3 and promote mitochondrial phagocytosis by autophagic vesicles, thereby eliminating lysosome-induced mitochondrial destruction. Moreover, mice with Bnip3 gene deletion exhibited some degree of inflammation and higher levels of ROS [84]. Impaired mitophagy has been associated with a variety of human diseases (e.g. MAFLD and tumors) [85]. Diminished or impaired mitophagy disrupts the production of healthy mitochondria and causes impaired mitochondria aggregation. In addition, mitophagy also has a function in activating inflammatory vesicles [86]. Moore et al. linked mitochondrial damage to increased severity of NAFLD in obese patients [87]. He indicated a 40-50% alleviation in ß-oxidation in individuals with NASH, which was linked with elevated hepatic ROS and decreased indices of mitochondrial biosynthesis, mitosis, autophagy, fission, and fusion. The study of mitophagy and NAFLD goes far beyond this. Macrophage-stimulated 1 (Mst1) is a novel upstream regulator of mitophagy that affects apoptosis in cancer cells by inhibiting mitophagy. Mst1 was found to promote NAFLD by disrupting Parkin-associated mitophagy. The specific mechanism is that Mst1 regulates parkin expression through the AMPK pathway. AMPK blockade inhibits Parkin-associated mitophagy and thus affects hepatocyte mitochondrial apoptosis [88]. In addition to this, mitosis is regulated by hormones. Thyroid hormones also reportedly alleviate NAFLD by enhancing FA oxidation, mitosis, and mitochondrial biogenesis [89, 90], as these hormones can increase mRNA expression of NIX, BNIP3, p62, ULK1, and LC3 under certain conditions to mediate mitophagy [91]. Mitochondrial bioregulation alters and coordinates the mitochondrial pool’s amount and quality between mitoses, allowing cellular mitochondrial activity regulation in response to cellular stress, metabolic status, and other intracellular hormonal and environmental signals and to induce mitophagy to complete the repair of diseased mitochondria to slow the progression of NAFLD.

### Oxidative stress

Mitochondria are organelles with complex structures and comprise OMM the IMM, and the mitochondrial matrix. Most mitochondria-associated proteins are produced in the cytoplasm and then transported to their site of action. The OMM is porous and allows the passage of ions and small uncharged molecules, while IMM consists of a complex of electron transport systems, transport proteins, and ATP synthases [92].

Energy is mainly produced in mitochondria in the form of ATP via pyruvate and FAs metabolism. To undergo β-oxidation, cytoplasmic FAs need mitochondrial entry [93], whereas, short- and medium-chain FAs can freely diffuse into the mitochondrial matrix. Long-chain Acyl-Coenzyme-A synthase in OMM activates long-chain FAs to Acyl-Coenzyme-A. On the outer side of the IMM, CPT1 transfers acyl groups from Acyl-Coenzyme-A to carnitine to form acyl-carnitine. Carrier protein carnosine-acylcarnitine translocase transports acyl-carnitine across the IMM. The mitochondrial matrix’s CPT2 converts acyl-carnitine to carnitine and Acyl-Coenzyme-A. Within the mitochondria, the β-oxidation cycle of four enzymatic steps degrades Acyl-Coenzyme-A [94]. In each cycle, Acyl-Coenzyme A shortens and two carboxy-terminal carbon atoms are released as acetyl coenzyme-A [95]. The first β-oxidation step is the dehydrogenation of acyl coenzyme-A to trans-2-enoyl-Coenzyme-A by Acyl-Coenzyme-A dehydrogenase. The last three steps are catalyzed by the mitochondrial trifunctional protein complex (MTP). In the second step, enoyl coenzyme-A hydratase catalyzes the hydration by producing (S)-3-hydroxy-Acyl-Coenzyme-A, which is then dehydrogenated by (S)-3-hydroxy-Acyl-Coenzyme-A dehydrogenase to generate 3-ketoacyl coenzyme A [39]. Lastly, thiolase cleaves 3-ketoacyl coenzyme-A to produce a two-carbon chain shortened Acyl-Coenzyme A and Acetyl-Coenzyme A. Aside from Acetyl-Coenzyme-A that enters ketogenesis and the TCA cycle, β-oxidation also generates nicotinamide adenine dinucleotide (NADH) and flavin adenine dinucleotide (FADH2). Electron Transport Chain (ETC) utilizes NADH and FADH2 to produce ATP. ATP is also generated by OXPHOS, which links the oxidation of NADH or FADH2 with the phosphorylation of ADP to form ATP [94].

**Fig. 4:**
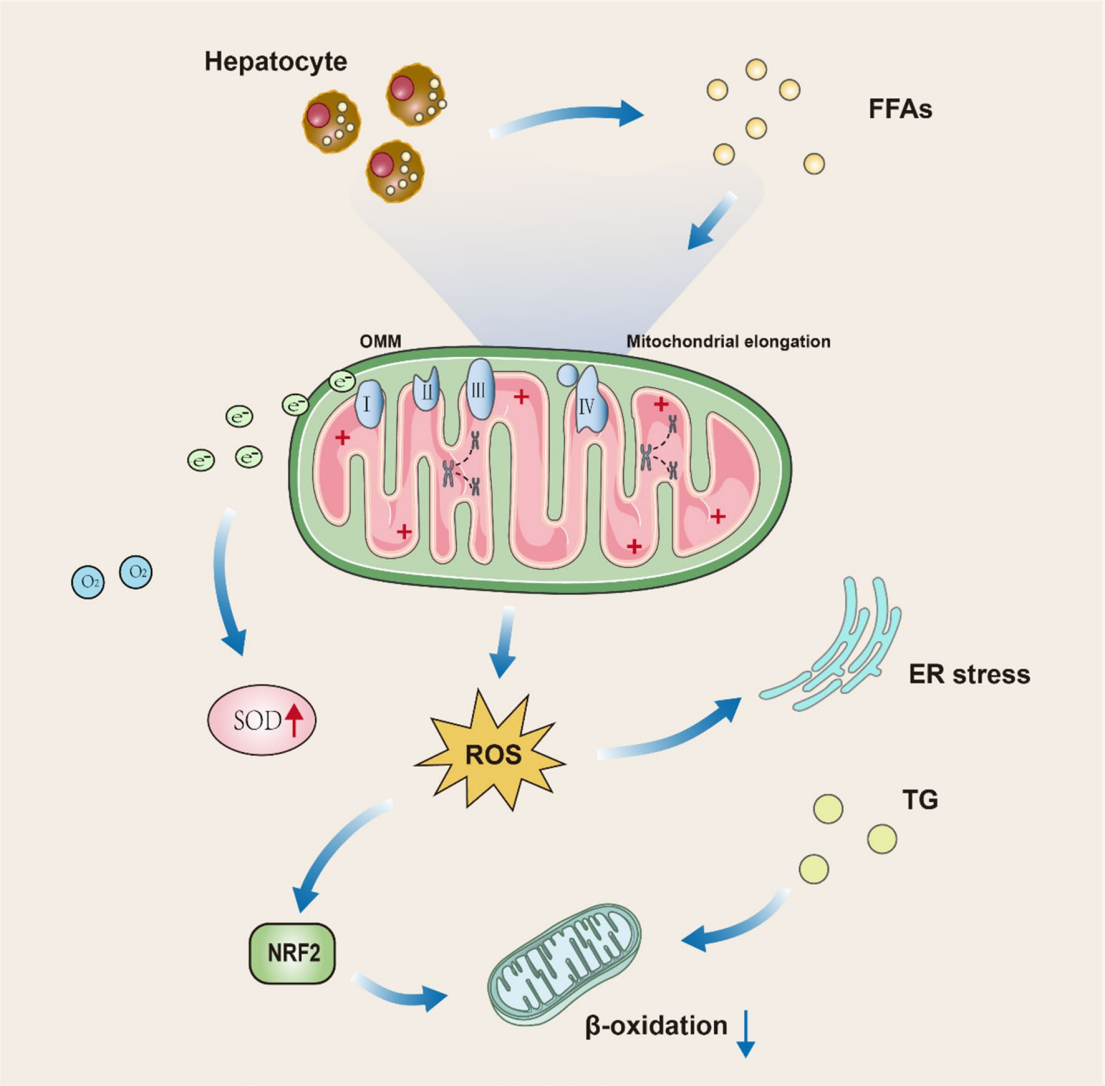
Specific flow diagram of oxidative stress. Specific flow diagram of oxidative stress. The large amount of free fatty acids produced by hepatocytes can act on the four oxidative respiratory enzymes of the mitochondria, leading to electron spillage. The spilled electrons combine with oxygen to form superoxide and cause the mitochondria to produce large amounts of reactive oxygen species. High levels of reactive oxygen species can lead to endoplasmic reticulum stress and reduce cellular beta-oxidation levels through stimulation of nuclear factor E2-related factor 2

Mitochondria produces 90% of cellular ROS [96, 97]. Fatty degeneration of the liver reduces the efficiency of the respiratory transport chain, generating ROS and ER stress (Fig. 4) [98]. Few electrons may escape ETC during ATP synthesis and react with oxygen to generate ROS. Under physiological conditions, only about 1–2% of mitochondrial oxygen loss produces ROS [99]. At this stage, electrons react with oxygen to form superoxide, which disrupts mitochondria by peroxidizing mtDNA [100], phospholipid Acyl chains, and respiratory transport chain enzymes [101]. In these conditions, ROS work as signaling molecules, and their production is countered by non-enzymatic and enzymatic antioxidant mechanisms. Additionally, increased lipid flow to hepatocytes dysregulates the de-phosphorylating activity of mitochondrial voltage-dependent anion channels and inner membrane permeability, causing mitochondrial depolarization, reduced ATP production, and loss of antioxidant activity [102–104], enhanced production of ROS [105, 106], and lipid peroxidation products (malondialdehyde (MDA) and 4-Hydroxy-2-nonenal (HNE) [107]. These processes then subsequently promotes apoptosis, inflammation, and liver fibrosis. Saturated FAs disrupt the mitochondrial membrane composition, favoring NAFLD progression [108]. In NAFLD, sustained FFAs flow and chronic acetyl coenzyme-A production can separate the function of the TCA cycle from mitochondrial respiration, producing excessive ROS production [109]. Excessive production of ROS causes oxidative damage to hepatocytes and exacerbates NAFLD [109, 110]. The release of ROS outside of liver cells causes hepatic stellate cells (HSCs) activation and extracellular matrix deposition. The relationship between ROS and NAFLD is much more than that. By analysing mitochondrial circular RNA (circRNA) expression profiles in fibroblasts with NASH, the investigators found that circRNA was down-regulated in a significant proportion. However, the construction of mitochondria-targeted nanoparticles that target circRNA SCAR can alleviate high-fat diet-induced cirrhosis and insulin resistance [111]. Excess liver FFA can induce the storage of toxic lipid intermediates, including ceramides [112]. Research suggests that the use of antioxidants to eliminate ROS from the cytoplasmic lysates and mitochondrial matrix can prevent simple steatosis and NASH [113]. Distinctive GPX1 deletion in hepatocytes protects mice from diet-associated NASH. However, there are few investigations show conflicting results [114–117]. The association of increased ROS production with enhanced detoxification and antioxidant activity of hepatic steatosis, but not with NASH, indicates that in NASH, the ROS overproduction mechanism may be inadequate [44]. As a continuous cycle sustained ROS release further damages hepatic tissue and dysregulates mitochondrial activity, thereby promoting fatty liver conversion to NASH in which mitochondrial adaptations are lost [118]. Indeed, ROS aggregation may activate c-Jun amino-terminal kinase (JNK) signaling and block the PPARα-FGF21 axis, thereby impeding mitochondrial β-oxidation and ketogenesis [119]. Chronic JNK stimulation is linked with apoptosis and chronic hepatic injury because it disrupts the function of the proto-oncogene non-receptor tyrosine kinase Src in the IMM, which is essential for the physiological activity of ETC [120, 121].

Decreases in the free hepatic radical scavengers, including mitochondrial MnSOD [45, 122], an increase in the oxidized form glutathione (GSH), and a reduction of glutathione disulfide (GSSG), also exacerbate oxidative stress. For instance, lower levels of MnSOD were similarly observed in humans, and in the liver of high-fat diet (HFD) fed rodents [44, 122]. In HFD-fed mice, accumulation of GSSG in the liver sensitizes hepatocytes to TNF-α-regulated cytotoxicity [123]. In addition, a study revealed that the level of both GSSG/GSH ratio and Glutathione-S-transferase (GST) were increased in the blood samples of 21 pediatric NASH children. Glutathione transferase is the second line of defense against oxidative stress. Simultaneously, increased electron donors from enhanced nutrient supply flood the mitochondria, for example, and create a high proton gradient at the IMM. In case the ATP synthase response to proton leakage fails to increase its activity, mitochondria may produce more ROS [124]. Compared to NAFLD and controls, obese NASH patients had the most enhanced levels of hydrogen peroxide (H_2_O_2_) and 8-OH-deoxyguanosine (8-OH-DG) in the liver, a marker of oxidative DNA damage. Thus, the imbalance between antioxidant defense and free radical growth creates a favorable environment for oxidative damage, inflammation, and fibrosis in hepatocytes, promoting lipid peroxidation of cell membranes, apoptosis, and ROS-mediated somatic mutations of nuclear and mtDNA.

### Mitochondrial quality control

Excess lipids in hepatocytes stimulate the production of both mitochondrial ROS production and FA oxidation. In a vicious cycle, impaired mitochondria lose their activity, leading to aberrant OXPHOS and increased ROS generation. Increased accumulation and uncontrolled mitochondrial ROS generation can harm mitochondrial components, including proteins, membranes, and mtDNA, and induce mitochondrial quality control (MQC) [125–127]. As an initial reaction to stress/ROS, MQC includes biogenesis, division, fusion, and mitosis, where mitochondria first try to retain their function and structure via DNA repair, antioxidants, protein folding, and degradation mechanisms. Mitochondrial biosynthesis, fusion, and division compensate for its activity. If the first response is ineffective, a more extensive MQC system is initiated [128, 129]. Impaired mitochondria can be improved by fusing with healthy mitochondria, however, severely damaged ones are isolated from healthy mitochondria by division and then targeted for degradation by mitophagy as described earlier [130, 131]. Endothelial nitric oxide synthase (eNOS) is a master regulator of mitochondrial quality control, and mice lacking eNOS are more susceptible to liver inflammation and fibrosis. A series of studies on liver mitochondria from eNOS knockout mice, among others, revealed that eNOS is an important regulator of liver mitochondrial content and function as well as NASH sensitivity [132]. MQC failure leads to loss of mitochondrial function and is among the potential causes of NAFLD progression [133].

## Therapeutic potential of improved mitochondrial function for NAFLD

So far, several therapeutic strategies have emerged in this field, but with different goals, as stated below. More evidence is needed to validate these treatments before they can enter clinical trials.

### Physical exercise

The current study shows that physical exercise stimulates the synthesis of mitochondria in the liver and transverse muscle [134]. Therefore, it can be hypothesized that physical activity can prevent and treat NAFLD by regulating mitochondrial activity and structure [135]. Studies have shown that an effective acute physical exercise session performed once has an impact on liver metabolism and redox status. However, whether there is permeability of protons in the mitochondrial membrane, 4th and enhanced 3rd state respirations, and stress response to mitochondrial permeability transition are affected needs further exploration [136, 137]. Prolonged physical activity for endurance training (or voluntary running) improves indices of the liver mitochondrial integrity and function and may promote a more stress-resistant or disease-resistant typical phenotype of the liver [137]. The restoration of mitochondrial function by physical exercise is not only seen in the liver. It was shown that in ovariectomised rats, exercise restored mitochondrial oxygen consumption and that even after ovariectomy, physical exercise compensated for the damage caused by ovariectomy by improving mitochondrial function [138].

The results of existing animal model studies have now presented an association between physical exercise and functional transformations of mitochondria. For example, Otsuka Long-Evans Tokushima Fatty (OLETF) rats developed obesity and T2D with multiple phenotypes of the metabolic syndrome [139, 140], the liver of OLEFT rats after daily voluntary running cycles for 16 or 36 weeks showed many changes including enhanced mitochondrial FA oxidation, oxidase function, and protein content. In addition, rat-based studies have also shown that levels of proteins associated with hepatic neolipogenesis are suppressed after physical exercise [141, 142]. Of course, this change parallels several indices of the mitochondrial OXPHOS apparatus, namely, increased citrate synthase, palmitate oxidation, β-hydroxyacyl-CoA dehydroacenaphthene, palmitoyl-CoA transferase 1 activity, cytochrome c, and ETC complex IV. Other effects include increased phosphorylated form of acetyl-CoA carboxylase (ACC) and decreased ACC, FA synthase, and activity of stearoyl-CoA desaturase (SCD) (inhibition markers of new hepatic lipogenesis) [143], further supporting the idea that physical exercise slows the progression of NAFLD by restoring normal mitochondrial biogenesis. In addition to this, exercise increases the oxidative capacity of liver mitochondria, which in turn improves the IR that drives hepatic steatosis. The mechanisms include enhanced FA oxidation and decreased synthesis of FA-derived ceramides and diacylglycerols associated with hepatic IR [144, 145]. Physical exercise and endurance training improve biogenesis and autophagy of mitochondria [146] and reduce mPTP opening in HFD-fed rats [147]. Daily physical activity is also linked with increased orthomorphic mitochondria in the liver, epigenetic modification of mtDNA (i.e., MT-ND6 hypermethylation), and improved severity of NAFLD [58]. In addition, physical exercise improves NAFLD by reducing intrahepatic fat content, increasing beta-oxidation of fatty acids, inducing hepatoprotective autophagy, overexpressing peroxisome proliferator-activated receptor gamma (PPAR-γ), and attenuating hepatocyte apoptosis and increasing insulin sensitivity. In conclusion, physical activity is closely related to the restoration of mitochondrial function and is essential for the treatment of NAFLD[148]. However, the low adherence to patients’ lifestyles has reflected the immediate medicine requirement [149].

### Anti-diabetes drugs

Peroxisome proliferator-activated receptors (PPARs) belongs to the superfamily of nuclear receptor that binds to a variety of FAs and their derivatives, including binding to several FFAs and their derivatives, and transcriptionally modulates intracellular metabolic pathways [150]. Current studies suggest the existence of three types of PPARs, which are classified as PPAR-α, PPAR-δ (also called PPAR-β), and PPAR-γ based on the ligand and PPARs tissue distribution. PPAR-α is expressed in the adipose tissue, liver, skeletal muscle, heart, and kidney, and is responsible for the regulation of lipid transport, gluconeogenesis, and the hormone fibroblast growth factor (FGF)-21. PPAR-α activation switches liver metabolism toward FA transport and β-oxidation [151], improve plasma lipids by alleviating TGs and enhancing high-density lipoprotein (HDL) cholesterol [152]. In animal models PPAR-α deficiency showed a parallel trend with worsening hepatic steatosis, however, activation of the receptor by typical PPAR-α agonists (e.g. fibrates) did not show a significant effect on NAFLD, although serum TGs were reduced [153]. Liver, skeletal muscle, and macrophages expressed PPAR-δ improved insulin sensitivity, reduced liver glucose production [154], elevated FA oxidation, and reduced activation of Kupffer cells [155]. Liver PPAR-δ carried an anti-inflammatory role in macrophages [155]. Activation of PPAR-δ reduces steatosis, but overexpression of PPAR-δ may have an impact on maintaining glucose levels. For example, high doses of PPAR-δ agonists exhibit reduced fasting insulin concentrations in the treatment of Rhesus monkeys [156]. Fortunately, Elafibranor shows unique effects as a PPARα/δ agonist, not only increases FA β-oxidation (PPARα activity) but also improves IR and inflammation [154]. Therefore, its distinct and efficient effects on hepatic mitochondria demand further research. PPAR-γ is majorly found in adipose tissues and modulates glucose metabolism, adipogenesis, and adipose tissue differentiation. Thiazolidinediones (TZDs) are PPARγ agonists that act as insulin sensitizers and antidiabetic agents, and recent studies have found that this family of drugs may also have potential against NAFLD (Table 1) [157–159]. For example, pioglitazone is effective in improving NASH to some extent [160, 161]. In addition to this, studies have shown that rosiglitazone also has the ability to improve NASH [162–164]. Notably, there may be mitochondrial targets of thiazolidinediones (mTOT) in animals that are able to act as mitochondrial membrane complexes to participate in pyruvate transport. However, in a mouse model with NASH, pioglitazone appears to reverse this steatosis in the liver [165, 166]. The specific reason for this may be related to the ability of TZDs to inhibit the entry of pyruvate into the TCA cycle [167]. In addition, a new PPARγ agonist named MSDC-0602 K targets mitochondrial pyruvate carriers while minimizing direct binding to transcription factors [168]. Of course, further proof is required to determine the function of novel agents in the treatment of NAFLD, such as selective PPARα modulators (fibrates, K-877), and PPARγ agonists (INT-131), PPARα/γ (DSP-8658), and PPARδ (HPP-593) [151].

**Table 1:** Drugs under study or approved for the treatment of MAFLD

Category	Name	Function	References
PPARα/γ/δ agonists	Elafibranor	Increased fatty acid beta oxidation, improves insulin resistance and inflammation	[154]
	Thiazolidinediones (TZD)		[157, 159]
	Pioglitazone Rosiglitazone	Improves mitochondrial function, protects pancreatic beta cells and increases tissue sensitivity to insulin	
	MSDC-0602K		[168]
Biguanide hypoglycemic drugs	Dimethylbiguanide Phenelzine	Targeted mitochondrial pyruvate carrier	[224]
	Buformin	Improved insulin sensitivity of liver and peripheral tissues	[225, 226]
GLP-1 receptor agonist	Liraglutide Exenatide Lisiratide	Enhancement of mitochondrial structure, attenuation of ROS production and promotion of autophagy	[227]
Mitochondria-targeted antioxidants	Ursodeoxycholic acid (UDCA) Elamipretide		
Decoupling agent	Aramchol	Affects mitochondrial electron transport	[196]
	Mito-quinone (Mito-Q) MitoVitamin-E (MitoVit-E)	Reduction of SCD1 and maintenance of cellular redox homeostasis	[198]
	Silymarin	Protects cells from peroxide-induced oxidative damage and apoptosis	[228]
	Controlled Release Mitochondrial Protonin (CRMP)	Regulation of thioredoxin and nitric oxide (NO) derivatives to reduce lipid peroxidation	[229, 230]
Intravenous injection of functional mitochondria	Mitotherapy	Liver mitochondrial uncoupling, improving liver fibrosis and liver protein synthesis Reduced lipid content, improve cellular redox balance	[216, 217]

The classic T2D treatment drug, dimethyl-biguanide, improves insulin sensitivity in the liver and peripheral tissues. Loading oleic acid in cultured HepG2 cells induced steatosis, and metformin was able to reduce steatosis and improve hepatocyte function [169]. Among the mechanisms are a decrease in oxidative stress impairment, and modulation of protein levels linked with the mitochondrial apoptotic pathway and its inhibition. Metformin also stimulates AMPK, which activates mitochondrial synthesis and FA β-oxidation, which are important for maintaining and promoting mitochondrial function [170].

Liraglutide is an acylated glucagon-like peptide-1 (GLP-1) agonist. In cultured hepatic HepG2 cells, it ameliorated NASH by inhibiting nucleotide-binding oligomeric structural domains, NOD‑like receptors family pyrin domain containing 3 (NLRP3), and focalization via mitotic activation [171]. Additionally, liraglutide improved NAFLD in HFD-fed mice by increasing mitochondrial synthesis, decreasing ROS generation, and elevating autophagy via the SIRT1/SIRT3 pathway [172].

### Targeted at SIRT3

Recombinant Sirtuin 3 (SIRT3) is a mitochondrial NAD+-dependent deacetylase that is important for regulating the activity of proteins related to cellular metabolism [173]. The SIRT3 gene encodes three isoforms, and the two long isoforms in mice are SIRT3 protein (M1 and M2) expressed in mitochondria. On contrary, the short-SIRT3 protein (M3) type is expressed on the cellular membrane and lacks N-terminal mitochondrial targeting signals. All isomers possess deacetylase activity, although they are distributed in different positions [174–176]. During fasting, SIRT3 upregulates β-oxidation and ATP generation [177], inhibits ROS, and enhances mitochondrial biosynthesis by peroxisome proliferators-activated receptor γ coactivator lalpha (PGC-1α) activation [178]. In contrast, in mice lacking SIRT3, mitochondrial proteins are hyperacetylated and impair mitochondrial function [179]. SIRT3 is a greatly expressed sirtuin in the liver of mice, it improves mitochondrial activity and NAFLD by modulating ketogenesis, β-oxidation, mitogenesis, and antioxidant response systems [179]. In human and mouse NAFLD models, however, SIRT3 is downregulated [180]. SIRT3 and PGC-1α can be mutually regulated, and both decreased in HFD-fed mice, this downregulation of SIRT3 caused mitochondrial proteins hyperacetylation and enhances fat storage and oxidative stress in the liver [181]. Exposure of SIRT3-deficient mice to an HFD further elevated this acetylated state of hepatic proteins, reduced the activity of respiratory complex III and IV, and exacerbated oxidative stress [182, 183]. Palmitate-induced lipotoxicity increases ROS production and hepatocyte death in SIRT3-deficient primary hepatocytes [184]. SIRT3 overexpression altered the inhibition of ATP synthesis via palmitate treatment [185]. In addition, this overexpression also inhibited the production of ROS. HFD in SIRT3 deficient mice exacerbates obesity, IR, hyperlipidemia, hepatic steatosis, and inflammation, however, adenovirus overexpressing SIRT3 rescued this phenotype [183]. In addition to its mitochondrial effects, hepatic SIRT3 deficiency exacerbated hepatic steatosis in HFD mice by upregulating proteins associated with FA uptake, including CD36 and VLDL receptors [184].

SIRTs are a potent therapeutic NAFLD target because they provide protection to hepatocytes from the effects of lipotoxicity [186]. Small molecule sirtuin modulators have been developed, but a few compounds targeting human SIRTs are still in clinical development. The basic problem is to determine the isozyme specificity and site-specific delivery of SIRTs activators [187, 188]. Notably, SIRT4 may be associated with coronary artery disease (CAD) in obese patients with NAFLD and normal or slightly elevated liver enzymes [189]. Low levels of SIRT4 produce large amounts of ROS while regulating free fatty acids, which, together with the release of free fatty acids from adipose tissue breakdown, leads to endothelial cell dysfunction, resulting in the development of CAD.

### Bile acids affect mitochondrial function

In bile acids (BA), the naturally present “tertiary” dihydroxy ursodeoxycholic acid (UDCA), exterior of chenodeoxycholic acid (CDCA), has shown multiple liver protecting effects and improved liver conditions in individuals with multiple chronic liver disorders [190]. On the basis of previous data, UDCA is also being examined in NASH individuals. Previously in an open-label pilot investigation, UDCA demonstrated some beneficial effects on hepatic enzymes and degree of steatosis (at biopsy) in NASH patients [191]. At the mitochondrial level, lipophilic BAs, such as deoxycholic acid (DCA), CDCA, and lithocholic acid (LCA), suppressed the ETC. The effect of high concentrations of BA (100 µmol/L) on the IMM of intact mitochondria was nonspecific, whereas the effect in broken or intact mitochondria propagated with low BA concentrations (10 µmol/L) was specific (damage to complex I and III) [192]. In the liver condition of biliary stasis with excessive BA retention, the antioxidant capacity of mitochondria is reduced [193]. In addition, cholestasis is associated with impaired mitochondrial function. At toxicologically suitable levels, most (but not all) BAs changes mitochondrial bioenergetics [194]. UDCA shows antioxidant and anti-inflammatory properties and prevents mitochondrial dysfunction in the progression of obesity-related diseases. In addition to this, it has been found that UDCA and Tauroursodeoxycholic Acid (TUDCA) as well as a lipophilic BA (CDCA and LCA) in mitochondria isolated from rat liver can have some effect on ETC function [195].

Aramchol (Arachidyl-amido cholanoic) also demonstrated potential effects in humans on hepatic steatosis, however, failed to improve glucose metabolism, hepatic enzymes, or insulin sensitivity [196, 197]. Aramchol in animal models ameliorates fibrosis and steatohepatitis by decreasing stearoyl coenzyme-A desaturase 1 (SCD1) and elevating fluxes that maintain cellular redox homeostasis through the transsulfur pathway [196]. Mice deficient in SCD1 have reduced lipid synthesis, increased mitochondrial FA β-oxidation, and insulin sensitivity in different tissues, including hepatic tissues. Therefore, SCD1 insufficiency has been associated with the prevention of hepatic steatosis in several nonalcoholic fatty liver mice models, such as high carbohydrate and HFD mice.

### Mitochondria-targeted antioxidants

Several antioxidants targeting mitochondria exist, but their specific role in the clinical arena needs to be further explored. Mito-quinone (Mito-Q) and MitoVitamin E (MitoVit-E) are potential new antioxidant classes. Both molecules contain covalently linked lipophilic triphenylphosphine (TPP) cation molecules, which allow them to pass across and accumulate within the mitochondria [198–200]. Mito-Q ameliorated the metabolic syndrome in 8 weeks of HFD rats [201] and revealed enhanced hepatic mitochondrial cardiolipin levels and central phospholipid synthase expression [202]. Mito-Q can prevent increased cholesterol, TG, glucose, and mtDNA damage by oxidation, and hepatic steatosis in atherosclerosis and metabolic syndrome experimental models [203, 204]. Low Mito-Q and MitoVit-E doses provide protection to cells against peroxide-stimulated apoptosis and oxidative damage, in contrast to low concentrations of non-targeted antioxidants such as ubiquinone and Vit-E [205, 206]. The protective efficiency of MitoVit-E and Mito-Q may be regulated by the inactivation of caspase-3 and cytochrome-c release. Additionally, they alleviate ROS-triggered transferrin receptor-induced iron uptake, lipid peroxidation and peroxidation-induced complex I inhibition, and aconitase in mitochondria [206]. A phase II investigation in chronic hepatitis-C individuals revealed that Mito-Q decreased circulating levels of transaminase; indicating a reduction in hepatic inflammation and necrosis in these individuals [207].

Silymarin is the main compound extracted from silymarin (Silybum marianum). Silymarin has few hepatoprotective activities [208] and may improve IR, hepatic injury, and hepatic enzymes in NAFLD individuals [209, 210]. Silymarin phospholipid complexes comprising vit-E alleviated hepatic steatosis in NAFLD patients and markedly reduced hepatic fat infiltration in HFD rats [211]. This phenomenon may be achieved by modulating thioredoxin and derivatives of nitric oxide (NO), as well as by substantially reducing lipid peroxidation. Silymarin also improved mitochondrial alterations in the respiratory complex and protected subunit CII-30 of complex II [212].

Inhibiting the production of mitochondrial ROS by uncoupling is an effective strategy and an antioxidant approach. 2,4-dinitrophenol (DNP) is an artificial uncoupling agent with a potential for toxicity [213], however, further verification is needed regarding whether it can alleviate NAFLD [214]. Controlled release mitochondrial plasmin (CRMP) is an oral DNP formulation that induces a mild uncoupling effect in liver mitochondria. In a rat model, it can alleviate increased TG, IR, hepatic steatosis, and T2D [215]. In addition, CRMP normalized plasma transaminase levels, alleviated liver fibrosis, and liver protein synthesis, and showed no toxicity at a systemic level in a methionine/choline-deficient NASH rat model [215].

### Mitotherapy

Dispersion therapy is a procedure in which functional mitochondria from exogenous hepatocytes are administered intravenously. This process may be successful in ameliorating HFD-induced hepatic steatosis. Among the specific mechanisms include the reduction of lipid molecules and improvement of redox homeostasis in cells. With this approach, exogenous mitochondria can be labeled with a green fluorescent protein and reacquired in the lung, brain, liver, muscle, and kidney of mice [216, 217]. This approach reduces fat deposition, prevents cellular damage, and increases energy production while restoring liver cell activity. However, the mechanism by which intact mitochondria infiltrate various cells and resume cellular metabolic activities is still undetermined [218]. Currently, related studies are focused on mitochondrial metabolism, for instance, which metabolic and proteomic variations are present in mitochondria extracted from normal liver cells compared to those of non-tumor origin.

## Conclusions and future perspectives

For the moment, there is some literature on the relationship between NAFLD and mitochondrial function. However, according to the search results, this literature is more about the link between NAFLD and a certain function of mitochondria, such as aspects of mitophagy [12, 80], oxidative stress [118], etc. Or a single presentation on how to treat NAFLD through mitochondria, such as mitochondrial transplants [32], herbal medicine [118], etc. We found no literature that integrates the above and the focus of this article is on the structure and function of mitochondria and the various current therapeutic modalities regarding mitochondria, respectively, to give the reader a better understanding of the close link between mitochondria and NAFLD. Metabolic dysfunction-related fatty liver or NAFL diseases are now among the most widespread chronic liver diseases globally, with MAFLD expected to overtake viral liver disease and put people at increased risk of terminal-stage liver disease, HCC, and CVD. Environment, genetics, and metabolic dysfunctions may be the cause of the pathophysiological development of NAFLD, such as alterations in liver lipid constituents, cellular impairment, and tumorigenesis [219]. Mitochondrial aberration and oxidative stress are hallmarks of NAFLD. Although animal models and human studies are contradictory, there is growing evidence that mitochondrial cycling can fall into imbalance during NAFLD. In terms of the mechanisms responsible for the regulation of mitochondrial morphology and dimensions, recent investigations suggest that mitochondrial metabolism alterations may begin in the initial NAFLD stages [220], speculating whether it could be considered a mitochondrial disorder. In addition, mitochondrial abnormalities persist during the disease course and may contribute to the advancement of MAFLD to HCC and NASH [221]. Therefore, it is crucial to prevent and treat NAFLD at any possible stage.

Some new potent drugs and molecular targets have been determined for improving treatment outcomes. However, recent clinical trial results suggest that understanding the pathophysiology of NASH is still limited and is far from achieving an optimal therapeutic strategy. In addition to affecting the genetic, metabolic, or environmental risk factors and stressors, therapeutic approaches may require several fundamentally important subcellular organelles as targets [222]. Shortly, one might consider using combination therapy. To date, no drug treatment has been approved against NAFLD, however, lifestyle alterations, physical exercise, and weight loss can regulate oxidative stress and the life cycle of mitochondria. The establishment of mitochondria-based therapies, some of which have been examined in humans (e.g., vit-E), has provided efficient benefits for NASH and has emerged as an anti-cancer adjuvant [223]. However, they depict only a small fragment of mitochondrial metabolism that may be regulated. Thus, a deep knowledge of the molecular structure of mitochondrial plasticity remains in its infancy, but it may furnish new doors for future physiological compound development having the potential for clinical NAFLD treatment. Ideally, factors that alter disease could simultaneously act on mitochondrial activity and energy production, rather than only on intracellular lipid metabolism regulators. Overall, we still need to be equipped with sufficient time and power to determine the prolonged efficacy and safety of each promising treatment option.

## Data Availability

Not applicable.

## Abbreviations

8-OH-DG: 8-OH-deoxyguanosine
ACC: Acetyl-CoA carboxylase
AD: Alzheimer’s disease
AKAP1: A kinase anchoring protein 1
AP-1: Activator protein-1
BA: Bile acids
BCL-2: B-cell lymphoma-2
BNIP3: Bcl2/adenovirus E1B 19 kDa interacting protein 3
CAD: Coronary artery disease
cAMP: Cyclic adenosine monophosphate
CDCA: Chenodeoxycholic acid
circRNA: Circular RNA
CRMP: Controlled release mitochondrial plasmin
CVD: Cardiovascular disease
DCA: Deoxycholic acid
DNL: De novo lipogenesis
DRAK2: Death associated protein related apoptotic kinase 2
Drp1: Dynamin-related protein 1
EGR: Early growth response
eNOS: Endothelial nitric oxide synthase
ER: Endoplasmic reticulum
FADH2: Flavin adenine dinucleotide
FAs: Fatty acids
FFA: Free fatty acids
FGF: Fibroblast growth factor
FIS1: Fission protein 1
GFP: Green fluorescent protein
GLP-1: Glucagon-like peptide-1
GSH: Glutathione
GSSG: Glutathione disulfide
GST: Glutathione-S-transferase
GTP: Guanosine triphosphate
GWAS: Genome-wide association studies
H2O2: Hydrogen peroxide
HCC: Hepatocellular carcinoma
HD: Huntington’s chorea
HDL: High-density lipoprotein
HFD: High-fat diet
HNE: 4-Hydroxy-2-nonenal
HO-1: Heme oxygenase-1
IL-18: Interleukin-18
IL-1β: Interleukin-1β
IMM: Inner mitochondrial membrane
JNK: c-Jun amino-terminal kinase
LCA: Lithocholic acid
LIR: LC3 interaction region
MAFLD: Metabolic dysfunction-associated fatty liver disease
MAMs: Mitochondria-associated endoplasmic reticulum membranes
MERS: Mitochondrial-endoplasmic reticulum contact
MFF: Mitochondrial fission factor
MFN1: Mitofusin 1
MFN2: Mitofusin 2
Mito-Q: Mito-quinone
MitoVit-E: MitoVitamin E
MQC: Mitochondrial quality control
Mst1: Macrophage-stimulated 1
mtDNA: Mitochondrial DNA
MT-ND6: Mitochondria-encoded NADH dehydrogenase 6 (MT-ND6)
MTP: Mitochondrial trifunctional protein complex
NADH: Nicotinamide adenine dinucleotide
NAFL: Non-alcoholic fatty liver
NAFLD: Non-alcoholic fatty liver disease
NASH: Nonalcoholic steatohepatitis
NFκB/Orai1: Nuclear factor kappa-B/calcium release-activated calcium modulator 1
Nix: Nip3-like protein X
NLRP3: NOD‑like receptors family pyrin domain containing 3
NO: Nitric oxide
NRF2: Nuclear factor E2-related factor 2
OLETF: Otsuka Long-Evans Tokushima Fatty
OMM: Outer mitochondrial membrane
OPA1: Optic atrophy 1
OXPHOS: Oxidative phosphorylation
PARL: Progerin-associated rhodopsin-like
PD: Parkinson’s disease
PINK: PTEN-induced putative kinase 1
PKA: Protein kinase A
PNPLA3: Patatin-like phospholipase domain-containing 3
PPAR-γ: Peroxisome proliferator-activated receptor gamma
PS1: Presenilin-1
ROS: Reactive oxygen species
S1R: Sigma-1 receptor
S637: Serine 637
SCD: Stearoyl-CoA desaturase
SIRT3: Recombinant Sirtuin 3
SREBP1c: Sterol regulatory element binding protein 1c
T2D: Type 2 diabetes
TC: Total cholesterol
TG: Triglycerides
TGS: Triglycerides
TM6SF2: Transmembrane 6 superfamily member 2
TNF-α: Tumor necrosis factor-α
TPP: Triphenylphosphine
TUDCA: Tauroursodeoxycholic acid
TZDs: Thiazolidinediones
UDCA: Ursodeoxycholic acid
VLDL: Very low density lipoproteins
